# Use of access port covers in transport incubators to improve thermoregulation during neonatal transport

**DOI:** 10.1038/s41598-023-30142-9

**Published:** 2023-02-23

**Authors:** Takahiro Fukuyama, Takeshi Arimitsu

**Affiliations:** Department of Pediatrics, Keio University School of Medicine, 35 Shinanomachi, Shinjuku-ku, Tokyo 160-8582 Japan

**Keywords:** Paediatric research, Techniques and instrumentation, Neonatology

## Abstract

Hypothermia in newborns increases the risk of health complications and mortality. This study aimed to evaluate the effectiveness of using covers over snap-open access ports of a transport incubator to maintain the temperature within. The change in temperature inside the transport incubator was evaluated over a 15-min period at three ambient room temperatures (20 °C, 24 °C, and 28 °C), as well as for three snap-open access port conditions: closed, where ports are closed; open, where the two ports on one side are open; and covered, where the two ports on one side are open but a cover is used. The automatic temperature control of the incubator was set to 37 °C for all conditions. We repeated the same experiments three times. The temperature decrease inside the incubator was greater for the open than for the closed or covered access port conditions at all three 4 °C-increasing room temperatures (*p* < 0.05). The incubator temperature decreased as a function of decreasing room temperature only for the open condition, with no significant difference between the closed and covered conditions. Therefore, snap-open access port covers provide an option to maintain a constant temperature within the transport incubator, which may lower the risk of neonatal hypothermia.

## Introduction

Hypothermia in newborns in the neonatal intensive care unit (NICU) increases the risk of health complications and mortality and, thus, must be prevented^1–3^. In particular, a 1 °C decrease in body temperature on admission to the NICU is associated with a 28% increase in mortality and an 11% increase in the probability of late-onset sepsis for extremely low-birthweight neonates^4^. Additionally, hypothermia can cause acidosis, hypoglycemia, and hypoxia^5^.

Very low-birthweight neonates easily decrease their body temperature. First, they lack a heat production mechanism. For example, they cannot shiver, do not have enough vascular control, and have low glycogen levels and brown fat storage^6,7^. Second, they easily lose heat because they have a relatively large body surface area with thin subcutaneous tissue necessary for thermal insulation^6,7^. Therefore, it is more important to maintain the body temperature while transporting very low-birthweight neonates.

In the first 10–20 min at birth, no intervention to prevent neonatal heat loss may make the neonatal body temperature fall by 2–4 °C^7^. Therefore, various measures are used in the NICU to prevent neonatal hypothermia, including control of environmental temperature; use of warm blankets, plastic wrapping, caps, and thermal mattresses; and skin-to-skin care^8,9^.

The World Health Organization and a 2013 randomized controlled trial recommended an ambient temperature of 25 °C for delivery and operating rooms used for neonatal care^10,11^, with temperatures of 23–25 °C recommended in the 2020 International Consensus on Cardiopulmonary Resuscitation and Emergency Cardiovascular Care Science with Treatment Recommendations^8^. The European Resuscitation Council 2021 Guidelines make more specific recommendations for ambient temperatures of 23–25 °C for neonates of 28–32 weeks of gestation and > 25 °C for neonates of ≤ 28 weeks of gestation^12^. Despite these guidelines, neonatal hypothermia at the time of admission to the NICU remains an issue of concern in practice^1,2,13–16^. Therefore, more reliable methods to prevent neonatal hypothermia are needed^17^, including during transportation to the NICU.

Inadequate warming during transport from the delivery or operating room to the NICU through cool ambient-temperature passageways is a potentially modifiable cause of hypothermia upon admission to the NICU^14^. To date, however, strategies to prevent neonatal hypothermia during transport to the NICU have not been comprehensively considered. This issue becomes specifically important when the snap-open access ports of an incubator are opened during transport to provide bag-valve-mask ventilation to the neonate or to hold the intubation tube. We hypothesized that attaching covers to the snap-open access ports could improve airtightness and thus prevent a drop in temperature within the incubator during transport. Therefore, the aim of this study was to evaluate the effects of three conditions of airtightness of the snap-open access port (open, closed, and covered) on temperature control within a transport incubator. To the best of our knowledge, this is the first study to consider these incubator-specific effects on the risk of neonatal hypothermia during transport.

## Methods

### Statement of ethics

Ethics approval was not required for our study as there was no involvement of humans or animals.

### Description of the set-up

**Figure 1:**
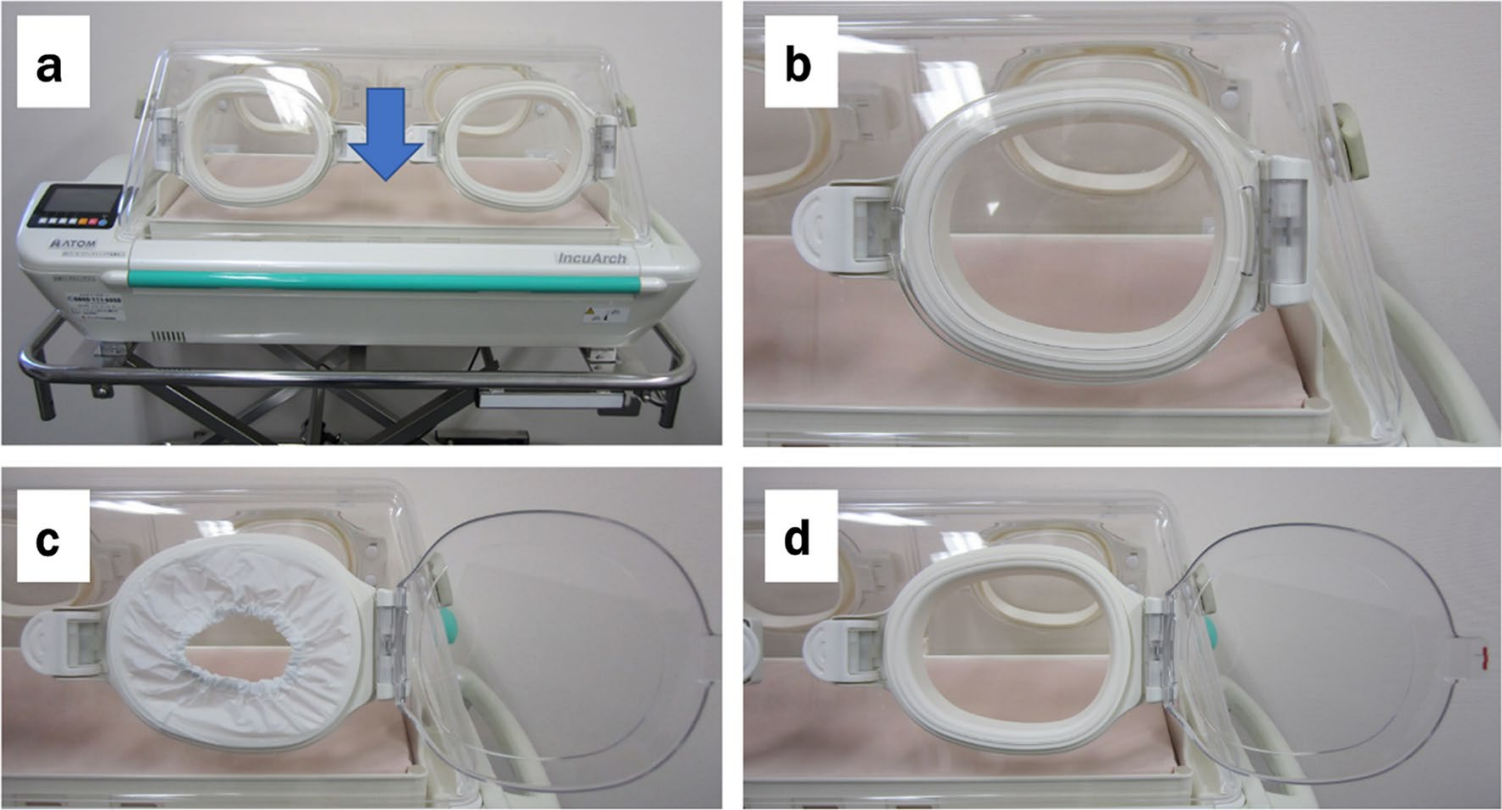
Pictures of the transport incubator. (**a**) Overall view; (**b**) the snap-open access ports are closed (“closed” condition); (**c**) the two snap-open access ports on the same side are opened, with covers attached (“covered” condition); and (**d**) the two snap-open access ports on the same side are opened, without covers attached (“opened” condition). Pictures taken by Takahiro Fukuyama.

The ATOM IncuArch^®^ (Atom Medical, Tokyo, Japan) transport incubator was used (Fig. 1). The incubator was placed in a closed space, with the room temperature maintained constant at three temperatures (20 °C, 24 °C, or 28 °C), using an air conditioner to lower the temperature as necessary. The room temperature and humidity were monitored using a digital thermometer (HMI41 indicator, HMP45 Humidity and Temperature Probe^®^; Vaisala, Vintaa, Finland), calibrated by the manufacturer. The temperature control inside the incubator was set to 37 °C and monitored using a thermometer (Incu I Tester^®^, Atom Medical) calibrated by the manufacturer and placed at the center of the mattress (arrow in Fig. 1a). We did not measure the flow ventilation, but during the experiments, the doors and the windows of the experimental room were tightly closed, no one entered the room, and we did not move inside the room to reduce the effect of airflow.

### Experimental conditions

The effects of the following three snap-open access ports conditions on the temperature inside the incubator were evaluated (Fig. 1): snap-open access ports are closed (“closed”; Fig. 1b); the two snap-open access ports on the same side of the incubator are opened with covers attached (“covered”; Fig. 1c); and the two snap-open access ports on the same side are opened without covers attached (“opened”; Fig. 1d). The effects of these conditions were evaluated for the 4 °C-increasing room temperatures used (20 °C, 24 °C, and 28 °C). The temperature inside the incubator was measured every 2 s over a 15-min period for each of the nine conditions (three temperatures for each of the three access port conditions), with temperatures at the start and end of this period recorded for analysis. We decided the observation time to be 15 min because we assumed that transport from the delivery room to the NICU was 15 min, as reported in a previous study^18^.

We conducted three experiments for each combination of port conditions and room temperature. The number of combinations was nine; thus, the number of experiments was 27. We determined the sample size by setting the *p* value to 0.05, effect size to 0.7, and power (1 − β error probability) to 0.8.

### Statistical analysis

A Shapiro–Wilk test confirmed the normal distribution of the data for the nine conditions. Between-condition differences were evaluated using a two-way analysis of variance, with the temperature inside the transport incubator as the dependent variable and room temperature and snap-open access port condition as fixed factors. The Bonferroni test for multiple comparisons was used to evaluate interactions between room temperature and access port conditions. Significance was set at a *p* value < 0.05 for all analyses.

## Results

### Descriptive analysis of measured room temperatures and room humidities

The room temperatures were consistent throughout the period of measurement, with the mean and standard deviation of the start and end temperatures as follows: room temperature of 28 °C, 28.06 °C ± 0.09 °C, and 28.08 °C ± 0.12 °C (*p* = 0.559); room temperature of 24 °C, 24.01 °C ± 0.11 °C, and 24.02 °C ± 0.19 °C (*p* = 0.760); and room temperature of 20 °C, 20.37 °C ± 0.21 °C and 20.43 °C ± 0.21 °C (*p* = 0.397), respectively.

The room humidities were measured at the start and end of the experiments, with the median and interquartile range of the start and end humidities as follows: room humidity of room temperature 28 °C, 42.0% (40.0–59.0%), and 41.9% (41.0–60.9%); room humidity of room temperature 24 °C, 51.2% (50.2–53.6%), and 50.8% (50.6–53.0%); and room humidity of room temperature 20 °C, 54.3% (54.0–56.3%) and 55.0% (53.8–56.2%), respectively. There is no significant difference between the three room temperature conditions (start room humidity *p* = 0.115, end room humidity *p* = 0.150).

### Effects of access port conditions and room temperature

**Figure 2:**
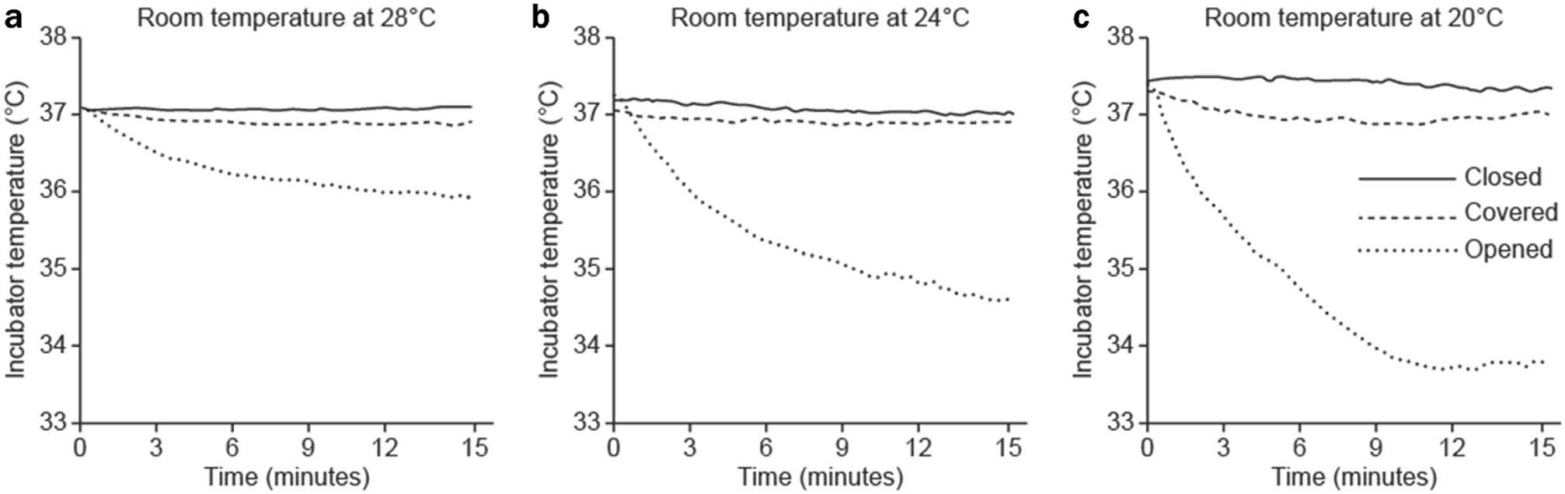
Representative graph of the temperature changes inside the transport incubator for the three snap-open access port conditions. Access port conditions are indicated as follows: solid lines, “closed” condition; dotted lines with wide spacing, “covered” condition; and dotted lines with narrow spacing, “opened” condition. The temperature inside the incubator at the start and end of the 15-min period of temperature measurement, respectively, for the three snap-open access ports conditions are as follows: (**a**) Room temperature at 28℃: “closed” condition, 37.05 °C ± 0.03 °C and 37.07 °C ± 0.05 °C; “covered” condition, 37.06 °C ± 0.03 °C and 36.06 °C ± 0.03 °C; and “opened” condition, 37.04 °C ± 0.03 °C and 35.88 °C ± 0.04 °C. (**b**) Room temperature at 24℃: “closed” condition, 37.08 °C ± 0.55 °C and 37.07 °C ± 0.17 °C; “covered” condition, 37.09 °C ± 0.15 °C and 36.99 °C ± 0.18 °C; and “opened” condition, 37.04 °C ± 0.03 °C and 35.01 °C ± 0.47 °C. (**c**) Room temperature at 20℃: “closed” condition, 37.34 °C ± 0.05 °C and 37.29 °C ± 0.04 °C; “covered” condition, 37.31 °C ± 0.03 °C and 37.07 °C ± 0.15 °C; and “opened” condition, 37.04 °C ± 0.03 °C and 33.46 °C ± 0.22 °C.

The temperatures inside the incubator at the start and end of the 15-min period of temperature measurement for the three snap-open access port conditions and the three 4 °C-increasing room temperatures are shown in Fig. 2. No significant differences were noted in the start temperature inside the incubator between the three access port conditions for all three room temperatures. However, an effect of room temperature (F (2,18) = 26.59, *p* < 0.001, η^2^ = 0.051) and access port condition (F(2,18) = 395.29, *p* < 0.001, η^2^ = 0.761) was noted on the end temperature within the incubator, with a significant interaction between these two factors (F(4,18) = 44.19, *p* < 0.001, η^2^ = 0.170).

**Figure 3:**
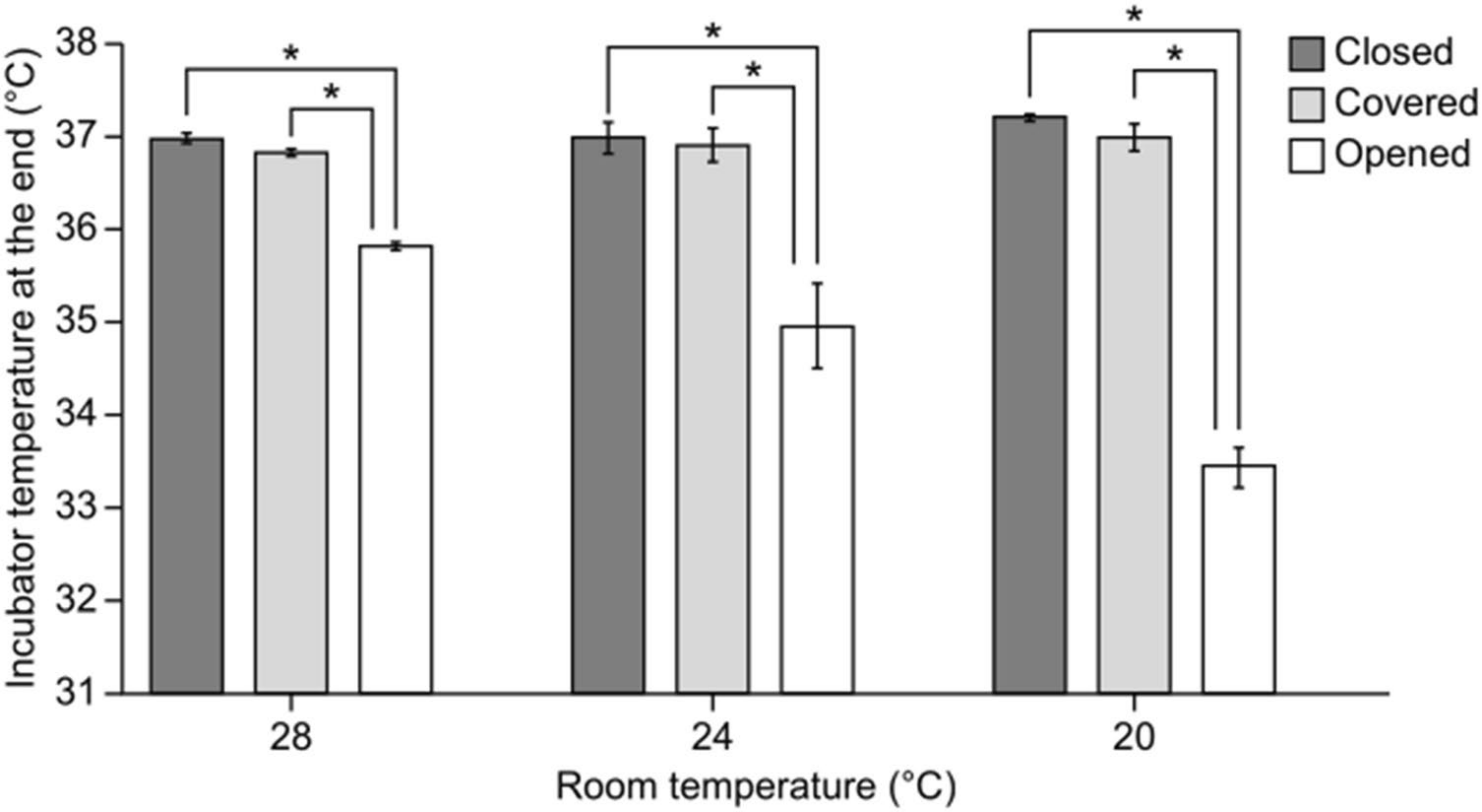
Comparison of end temperature in the transport incubator for the three snap-open access port conditions. The access port conditions are indicated as follows: dark gray bars, “closed” condition; light gray bars, “covered” condition; and white bars, “opened” condition. *Indicates statistical significance.

On multiple comparisons, a significant effect of room temperature was identified only for the “opened” access port condition (20 °C < 24 °C < 28 °C; *p* < 0.001). With regard to the specific effect of access port conditions on the end temperature within the incubator, the end temperature was lower for the “open” condition than for the “closed” condition (*p* < 0.001) and for the “open” condition than for the “covered” condition (*p* < 0.001) at all three increasing room temperatures, as shown in Fig. 3.

## Discussion

The use of access port covers was effective in preventing temperature decreases within the incubator, even for the cooler ambient temperature of 20 °C, which is below the temperature mentioned in the current guidelines for neonatal care environments^8,10–12^. We also showed that even if the ambient temperature is maintained at 28 °C, as recommended by various guidelines, the incubator temperature does decrease over a 15-min period when the access ports are opened^8,10–12^. As a means to reduce the risk of neonatal hypothermia, our findings indicate that the use of access port covers could prevent decreases in incubator temperature during transport when access ports must remain open to maintain ventilatory care to the neonate.

Maintaining the incubator temperature is clinically important to prevent neonatal hypothermia^1,2,13–16^. Our findings, considered together with those of previous studies on neonatal hypothermia, underscore the need for enhanced strategies for incubator temperature control, including the use of access port covers, minimizing the time during which access ports are open, and maintaining a higher ambient temperature in passageways. The importance of airtight access ports should be considered in the design of transport incubators and future guidelines^14^.

Transport incubator covers are reusable and very inexpensive, costing approximately USD $2.25 (about €2.25) each. Therefore, based on the results of this study, attaching covers to a transport incubator to prevent neonatal hypothermia at the time of admission to the NICU seems to be an economically feasible method from a healthcare perspective.

The limitations of our study need to be acknowledged in the application of findings to practice. First, the incubator was placed in a room, and thus the effects of motion during transport were not considered. Air convection inside the incubator during actual neonatal transport will affect the neonate’s body temperature. As airflow differs between facilities, experiments to evaluate general effects of air convection are difficult to conduct. Moreover, the ambient temperature or humidity changes between the delivery room, the passage to the NICU, and the NICU were not considered. Further research to investigate these changes should be conducted in the future. Second, in the “opened” access ports condition, we did not consider the effect of medical staff placing their arms through the port to provide care. This, in fact, could offer a benefit against temperature decreases when the access ports are open. Third, we decided the observation time to be 15 min because we assumed that transport from the delivery room to the NICU was 15 min, as reported in a previous study^18^. Further experiments with a longer observation time should be conducted if we assume a longer transport, such as the delivery between distant hospitals. However, we showed a significant incubator temperature difference between the port conditions during the 15-min observation period. Fourth, there may be some differences in the thermoregulation mechanism between manufacturers, thus, it is uncertain how temperatures within transport incubators change except for in the one manufactured by ATOM Medical Inc., which is the only one we used. However, regardless of the manufactures of incubators, the heat retention effect of the cover is expected by decreasing the extra space between the ports and the physicians’ arms. Further experiments with other transport incubators manufactured by other manufacturers are necessary. Moreover, to deepen the findings of this study, it is necessary to investigate how the airflow through the port changes depending on the presence or absence of the cover. If the airflow affects the incubator temperature, it may be desirable to have the cover on the port, as in this study, or to have a mechanism to decrease the airflow through ports. Fifth, we have not assessed the risks of access port covers. For example, the risk of neonatal infection can be increased by microorganisms transmitted through the cover. We must wash our hands up to the elbows as usual, although the covers can be disinfected. In addition, since the covers are made of very soft material, it does not restrict the movement of the medical staff's arms in them. However, if medical treatment is required for neonates during transportation, it may be difficult for the medical staff to move the arm. These risks in newborns should be evaluated in future studies. Finally, as a newborn was not placed in the transport incubator, we were unable to assess the effects on actual body temperature, and the effects of gestational weeks, birth weight, and congenital diseases of neonates; use of warm blankets, plastic wrapping, caps, and thermal mattresses; and skin-to-skin care on neonatal hypothermia are uncertain^8,9^. Owing to the risks of hypothermia on neonatal health outcomes and mortality, examining the effects of lower incubator temperature on body temperature is not feasible.

Despite these limitations, the findings of our study are valuable, as they show that the use of access port covers can be one of the feasible options to maintain the incubator temperature, which may result in lowering the risk of neonatal hypothermia during transport to the NICU, with other related factors. We also show that the incubator temperature cannot be maintained at the set value using only the automatic temperature control function of the transport incubator when the access ports are open without covering at least in 15 min.

In conclusion, snap-open access port covers can effectively maintain a constant temperature within the transport incubator, which may lower the risk of neonatal hypothermia during transportation. However, further research is necessary to check whether the snap-open access port covers lower the risk of neonatal hypothermia during transportation in a clinical setting to advance the future of neonatal medicine.

## Data Availability

The datasets generated and analyzed during the current study are available from the corresponding author on reasonable request.
